# Seeing life in the teeming world: animacy perception in arthropods

**DOI:** 10.3389/fpsyg.2024.1492239

**Published:** 2024-11-21

**Authors:** Massimo De Agrò, Hiruni Samadi Galpayage Dona, Giorgio Vallortigara

**Affiliations:** Center for Mind/Brain Sciences, University of Trento, Trento, Italy

**Keywords:** animacy, biological motion, face detection, invertebrate, praying mantis, jumping spider, bee, paper wasp

## Abstract

The term “animacy perception” describes the ability of animals to detect cues that indicate whether a particular object in the environment is alive or not. Such skill is crucial for survival, as it allows for the rapid identification of animated agents, being them potential social partners, or dangers to avoid. The literature on animacy perception is rich, and the ability has been found to be present in a wide variety of vertebrate taxa. Many studies suggest arthropods also possess this perceptual ability, however, the term “animacy” has not often been explicitly used in the research focused on these models. Here, we review the current literature providing evidence of animacy perception in arthropods, focusing especially on studies of prey categorization, predator avoidance, and social interactions. First, we present evidence for the detection of biological motion, which involves recognizing the spatio-temporal patterns characteristic of liveliness. We also consider the congruency between shape and motion that gives rise to animacy percept, like the maintenance of a motion direction aligned with the main body axis. Next, we discuss how some arthropods use static visual cues, such as facial markings, to detect and recognize individuals. We explore the mechanisms, development, and neural basis of this face detection system, focusing on the well-studied paper wasps. Finally, we discuss thanatosis—a behavior in which an animal feigns death to disrupt cues of liveliness—as evidence for the active manipulation of animacy perception in arthropods.

## 1 Introduction

### 1.1 What is animacy?

Life comes in innumerable forms. We gaze in wonder at the difference between the big marine mammals and the microscopic nematodes, between the disarticulated mollusks and the rigid coleopteran, or between the clever naked ape and the seemingly automatic rotifer.

Yet, for all of their differences, all animals share key commonalities. For example, most animals are characterized by a body symmetry, being it radial or lateral. For the latter, sensory organs are often concentrated at one extreme of the mirroring plane, which also often coincides with the heading direction during motion. To locomote, creatures activate rigid or semi-rigid extremities, capable of producing forward forces on the body thanks to a set of repeating and stereotyped movements. Even excluding these stereotypical structures and motion patterns, animals are the only beings in nature that possess the property of being “animated”: they can initiate or stop motion, change direction or speed, all without the intervention of external forces (Di Giorgio et al., 2016; Mascalzoni et al., 2010; Premack, 1990). With such wide commonalities, it should not surprise us that many sensory systems have evolved dedicated processing in order to detect and recognize animate entities, acting as “life detectors.” These sensory processes do not require any understanding of the “liveliness” property of the observed objects, which instead constitute the basis for more complex cognitive skills, like, for example, the theory of mind (Premack, 1990; Piaget, 1926). They instead may only rely on the perceptual cues that characterize the animated actors.

The ability of organisms to detect cues of “liveliness” in their environment (Lorenzi and Vallortigara, 2021; Vallortigara and Losi, 2021) is often referred to as “animacy perception.” With most of the research on the topic focusing on the visual modality, these cues are often related to specific body configuration (Kobylkov and Vallortigara, 2024), motion patterns (Johansson, 1973), or a combination of the two (Tremoulet and Feldman, 2000). The presence of this skill seems to be as widespread as the animacy cues are ubiquitous, from humans (Johansson, 1973) to non-human primates (Brown et al., 2010) and other mammals (Blake, 1993), birds (Lemaire and Vallortigara, 2022), fishes (Nakayasu and Watanabe, 2014), and mollusks (Mezrai et al., 2020).

### 1.2 Different scientists, different terminology

Given the widespread animacy perception found across the evolutionary tree, it is surprising that the literature on arthropods is rather scarce. This is probably due to the fact that, historically, animal cognition scientists have seldom extended their scientific inquiry to this Phylum, perceived to possess a brain too small and therefore cognitively limited to perform generalized computations. Yet, it has been proposed that while miniaturized nervous systems may be limited in memory capacity, they are sufficiently adept in the realm of cognition (Chittka and Niven, 2009; Vallortigara, 2025).

Today, the notion that arthropods are limited in their cognitive abilities is challenged by an expanding body of literature demonstrating their capacity for complex computation (Chittka and Niven, 2009; Bortot and Vallortigara, 2023). Animacy perception may in fact serve as an adaptive solution in small brains, allowing these organisms to tackle various challenges more efficiently using a generalized skill rather than multiple specialized ones.

Apart from a few direct studies, much of the research on prey categorization, predator avoidance, and social interactions in arthropods does imply the ability to detect life, but rarely uses the term “animacy perception.” This inconsistency in terminology makes it challenging to gather all relevant studies, as evidence is scattered across various fields. In this review, we present explicit and implied evidence of animacy perception skills, enabling individuals to detect static or dynamic cues in a wide range of arthropod species. What is presented here is by no means a complete list of all animacy cues available to arthropods (of which many more may exist, especially considering the wide arrangement seen in vertebrates. See Tsutsumi et al. (2012) for an example of an alternative cue), nor all the ones that they already are using. Instead, we will present the ones for which we have enough evidence to suggest, or at least discuss, the presence of a generalized life-detector.

## 2 Seeing life in motion

The ability to move is shared by all animated objects. However, not all motion is an effective cue of animacy, as also non-living objects can shift across the visual field if pushed, moved by a breeze, or dropped. It is the type of motion that can be indicative of whether we are observing a living agent or not. As stated above, animated objects are the only ones that can start and terminate their motion without the intervention of external objects, a property termed “self-propelledness” (Premack, 1990). The impression of liveliness given by this property is so potent that humans and other animals tend to interact socially with objects otherwise not resembling in any shape or form valuable companions (Di Giorgio et al., 2016; Mascalzoni et al., 2010; Premack, 1990). Yet, even without information about the start or end of an object’s trajectory, it is still possible to extract some other, finer characteristics of its motion pattern, that can act as a cue of the object’s animacy.

### 2.1 Biological motion

Animals that locomote display a specific spatiotemporal relationship between the different body parts, imposed by their body plans. The bodies of vertebrates and arthropods alike are in fact composed by linked, rigid segments, with the distance between interconnected joints (e.g., the wrist and the elbow in humans) remaining fixed for the whole duration of motion. Between other dots instead the relationship can vary, albeit still partially constrained by the general body plan (e.g., wrist to knee). Thus, when observed visually, the movements of these animals result in a statistically identifiable idiosyncratic pattern dubbed as biological motion (Johansson, 1973; Johansson, 1976). Thus, a stimulus perceived to be moving following this pattern can be assumed to possess animacy. Crucially, animals may extract this information even in displays completely devoid of structure. These stimuli are designed as clouds of dots (usually referred to as “point-light displays”) moving congruently with how the main joints of an animal would move. The rules governing the motion of the point-light displays are the same independently from the shape of the animal depicted, and as such, virtually universal for all animated objects. The perception of animacy is conserved even in “scrambled” point-of-light displays providing that the dots’ relative location is spatially randomized but with dots still moving biologically, i.e., In a semi-rigidly fashion (Troje, 2013; Troje and Westhoff, 2005; Vallortigara et al., 2005).

The ability to detect biological motion has been observed in many non-human animals (Blake, 1993; Regolin et al., 2000), but in arthropods it has only been described in jumping spiders (Arachnida: Salticidae) (De Agrò et al., 2021). These animals possess one of the most complex visual systems across arthropod species, unseen in the rest of the animal kingdom (Chong et al., 2024; Winsor et al., 2023). This is split among four different pairs of eyes, each projecting into dedicated brain structures (Steinhoff et al., 2020). This morphological segregation corresponds with a functional specialization, where the two anterior medial eyes (AME, also referred to as the principal eyes) are dedicated to figure discrimination (Strausfeld et al., 1993; Land, 1969). The other three pairs, anterior lateral, posterior medial and posterior lateral eyes (ALE, PME, PLE, also referred to as secondary eyes), are specialized in motion detection (Zurek and Nelson, 2012; Beydizada et al., 2024; Loconsole et al., 2024). This functional split between motion and shape detection in the jumping spiders’ visual system makes them an elective model for the study of biological motion displays, as the structural and temporal information that could be extracted from the point-light displays are detected from morphologically distinct sensory units. This in turn allows for the separate inquiry of these two types of information, and how they may interact for the detection of animacy.

De Agrò et al. (2021) tested the ability of the jumping spider *Menemerus semilimbatus* to discriminate biological from non-biological point-light displays. The authors fixated the spiders on an omnidirectional treadmill, and then presented it a pair of stimuli thanks to a computer monitor placed frontally. The stimuli pairings always presented a biologically moving stimulus (a point-light display moving like a spider, a scrambled version of it, or a moving spider silhouette) paired with a non-biological moving stimulus (a point-light display with the points moving randomly paired with the first two biological options, or a translating ellipse paired with the silhouette). Upon detection of a moving object in the visual field of the secondary eyes, the spiders perform a characteristic full-body pivot in order to face the target frontally and subsequently inspect it with the principal eyes. The authors recorded the differential tendency of the animals to pivot towards either the biological or the non-biological display. In every condition, the spiders showed a significantly higher tendency to pivot towards the non-biological displays, demonstrating their discrimination ability but showing an unexpected preference for the non-biological display.

In a subsequent experiment (De Agrò et al., 2024), the same authors selectively covered each of the spiders’ eye pairs with paint, in order to test which eye pair is responsible for biological motion discrimination, or if all of the secondary eyes are equally capable of solving the task. Using a similar procedure as described above, the authors observed that spiders with all eyes but the ALE covered showed a significant tendency to pivot towards the biological over the non-biological displays, reversing their preference with respect to the first study. Spiders with only the PLE available instead showed no preference, pivoting an equal amount towards biological and non-biological displays.

The results clearly demonstrate that spiders can discriminate biological and non-biological motion, and that this ability is specifically present in the ALE-connected brain circuitry. The authors suggest that this discrimination is based on a low-level detection system, where non-biological stimuli are not perceived at all by these eyes. PLEs seem instead to act as simple motion detectors, triggering pivots towards the stimuli but with no perceivable difference between the two. The concurrent activation of both ALE and PLE eyes can further inform decision making: a stimulus that can be computed by both pairs (i.e., biological motion) does not require further inspection, as it is appropriately categorized. In this context, it would be advantageous to turn towards the random stimulus, not recognized as animated and therefore requiring further inspection by the shape recognition eyes.

### 2.2 Biological motion with no limbs

As described above, the detection of biological motion patterns heavily relies on the detection of moving limbs, and their signature of semi-rigid motion (Johansson, 1973). Yet, many animals and especially many arthropods’ preys do not possess limbs: fishes, snakes, worms, caterpillars. Others do possess limbs but they are immaterial for locomotion: this is the case, e.g., of flying insects, where the motion trajectory is independent on the legs’ activity. Yet, all of these animals are alive, and do move in recognizable patterns. It is likely that arthropods may recognize animacy cues even in the absence of a classical biological motion pattern, as it happens in vertebrates (Rosa-Salva et al., 2016; Lorenzi et al., 2017; Lorenzi et al., 2021; Lemaire et al., 2022).

Bartos (2022) tested the predatorial decision making process of the jumping spider *Yllenus arenarius.* These arachnids occupy a very specific ecological niche, as they live exclusively in dunes (Bartos, 2013). They are opportunistic predators, stalking on many different insects, including caterpillars. Crucially, they employ different hunting strategies depending on the detected prey (Bartos, 2013). The author (Bartos, 2022) presented *Y. arenarius* spiders with various virtual preys, projected onto a white canvas inside the animal’s chamber. All of the stimuli presented an elongated gray rectangle, simulating a worm-like body. The body could either be long or short, and could be either crawling, simulating a caterpillar, or simply shifting in space. The author observed that the crawling movement alone, independently from body length, induced the spiders to engage with the virtual prey using their caterpillar-specific hunting strategy, with a frequency not different from real-life preys. This demonstrates that these spiders do rely on the worm-like motion to detect a possible prey, while it remains unknown whether this would suffice as an animacy cue: the spiders attacked also the non-wriggling preys, which may suggest that this type of motion is not an animacy cue, but just a specific hunting trigger.

More evidence comes from a different arthropod species, the praying mantis (Insecta: Mantodea). These animals are sit-and-wait predators and use vision as their primary sense. Often static on tree branches, they erupt in a quick extension of their forelimbs upon detection of a moving target, catching the prey between the spiked tibia and femur (Prete, 1999). While mantises do possess a fairly high visual acuity for an arthropod (Kral and Prete, 2004), the attack decision must happen in a fraction of a second, making the use of motion-based cues much most relevant. Yamawaki (2003) tested the attack probability of mantises when presented with virtual preys. The stimuli were non-translating but were instead composed of six interconnected circles, moving up and down, always maintaining a realistic, “whole body” structure. The pattern was used to simulate the movement of a caterpillar, lifting its head while maintaining its back attached to the substrate. The author found that both the elicited attention and the probability of attack by the mantises increased as a function of the amount of displacement of the wriggling “head.” Crucially, however, when the artificial caterpillar moved synchronously both the “head” and the “back,” the probability of attack dropped. This demonstrates that the amount of motion alone is not sufficient to elicit a response from the mantis, but instead this needs to be congruent with the natural motion of living organism. This is not the only possible explanation: the author suggested that synchronous displacement of the two caterpillar extremes may be perceived as two separate objects, inhibiting the hunting response by causing attentional conflict in the mantis. In a previous study, however, Yamawaki (2000) tested praying mantises with a different set of moving, non-translating stimuli. Rather than being caterpillar-like, these stimuli were composed of a square or rectangular body, with two sticks at the left and right side, moving up and down simulating legs motion. Here the author observed that the attack probability varied according to the presence of moving sticks and the size and orientation of the rectangular body. However, the distance between the two moving legs had no effect, casting doubt on the attentional conflict hypothesis.

### 2.3 The right motion for the body

The examples provided in the previous paragraph all described animacy cues primarily as motion-based. Animacy cues can however arise from the interaction between the body structure of the organism and its motion pattern, even when neither cue would elicit animacy perception alone.

Bilaterians are characterized by a single plane symmetry and are often elongated along that plane (Knoll and Carroll, 1999). Often, also their motion direction follows the same symmetry plane. Moreover, the direction of motion is also virtually exclusively congruent with the head positioning, usually placed on one of the two end points along the main body elongation axis. Thus, when in locomotion most animals move congruently with their elongated body axis, or/and in the direction of the head positioning, thus providing a general and effective animacy cue. Humans are particularly sensitive to this effect: we find objects that move sideways “odd,” and less “life-like,” while we report a clear sense of animacy when they move aligned to their principal axis (Tremoulet and Feldman, 2000). As the reader may expect by now, the ability to infer animacy from this body-motion orientation congruency is widespread across vertebrates (Cooper, 1981; Apfelbach and Wester, 1977; Rosa-Salva et al., 2018; Rosa-Salva et al., 2023). In arthropods, this has been studied mainly in the context of predatory behavior.

Being visually guided predators, it is no surprise that jumping spiders use the body-motion animacy cue to decide what is worthy of an attack. Bartos and Minias (2016) presented *Y. arenarius* spiders with various virtual preys, projected onto a white canvas inside the animal’s chamber. All of the stimuli presented an elongated gray rectangle, simulating a worm-like body. For some stimuli, the authors added a black circle (and for some stimuli also other details, like legs or antennae) on either side of the worm-like figure, simulating a head spot. When presented to the spiders, the stimuli could either (i) move along their main axis in the direction of the head; (ii) move along their main axis, but with the head trailing rather than leading (i.e., backwards); (iii) move up and down, and therefore at 90° in respect of their main axis. Unfortunately, the authors only report the detailed behavior of the spiders that finally decided to attack, rather than how many did so out of the total number of tested individuals. However, the authors found that the spiders almost exclusively hit the black spot when this was leading in the motion direction, while they instead chose at random between the black spot and the other end of the worm-like figure when this was trailing. They also observed that in this second configuration around half of the spiders engaged in front-rear observation (looking alternately between the two extremes of the stimulus) before attacking, while they rarely did so when the head position and motion were congruent. These results clearly show that *Y. arenarius* heavily rely on animacy cues in predatory decision making, attaching a high confidence value to the body-motion congruency, so much that violation of some of its characteristics in an otherwise alive-looking stimulus causes further information seeking attempts.

Jumping spiders are, however, not the only arthropods sensitive to the body-motion congruency. Prete and colleagues have studied which motion characteristics trigger stalking and attack in mantises, using visual stimuli on screens that varied in contrast, size, color, pattern, and speed (Prete et al., 2013; Prete and Mahaffey, 1993; Prete et al., 2008; Prete et al., 2011; Prete et al., 2012; Prete et al., 2013). Their research produced a detailed psychometric function showing hunting triggers that likely help the predators to differentiate between living prey and inanimate objects like a leaf moved by the wind. Among all of these motion characteristics, the authors tested the preference of mantises for objects moving following their main body axis. They observed that objects moving along their main body axis, with respect to ones moving perpendicularly to it, elicited significantly more stalking bouts and more attacks.

In this paragraph, we presented a wide range of cues that arthropods use while hunting for their prey. It appears that the amount of overall motion alone is insufficient to trigger an attack. They instead rely on varyingly complex motion characteristics, being them the movement of limbs or the whole body, with each element increasing the chance that the observed object is, in fact, alive. While the hunting context is traditionally not the focus of animacy perception in vertebrate studies, the motion characteristics described in this paragraph closely resemble the ones described in the wider literature on animacy (Troje, 2013; Vallortigara et al., 2005; Rosa-Salva et al., 2016; Rosa-Salva et al., 2018; Chang and Troje, 2008; Simion et al., 2008), suggesting that the basic mechanism is independent of the behavioral context and available to be exploited by many different animals in many in different tasks.

## 3 Seeing life in shapes: the case of face detection

Even if prominent, motion is not the only cue that can indicate the presence of an animated object. Indeed, motion information may be at times unavailable as an animacy cue. For example, predators are dangerous even while static, as it may be too late while they are in motion. Similarly in the social context, it is useful to direct attention to companions also when they are not locomoting, and maybe pick up on finer behaviors or facial expressions. These static cues are generally not as reliable as motion ones: for example, a dead predator or conspecific may look fully alive, while the lack of movement generally attests to the loss of animacy. However, these static cues remain fundamental in an initial evaluation of surrounding stimuli, and may act as catalysts of attention.

As stated in the introduction, animals often “look-alike,” as the common evolutionary origin often brings shared structures and patterns. For example, many living organisms present most of the sensory organs collected around the same spatial location (i.e., the head), and often in a common configuration: more eyes above a single mouth. It is no wonder that many animals have evolved dedicated circuitry to detect face-like images. Detecting a face involves seeing a specific triangular configuration as simple as two horizontally aligned dots (the eyes) and a third dot below (the mouth). It is crucial to point out that being capable of recognizing individuals may be achieved even without any face-dedicated brain area. For example, an efficient strategy to discriminate between faces may be to simply learn some particular aspects of it, i.e., by feature learning. Under this perspective, faces may be treated like just another visual stimulus, that can be learned using a generic circuitry. In the context of animacy perception, the key is the common top-heavy configuration of “face-like” stimuli, which allows for these to be categorized together, and computed by a dedicated brain area. To recognize the entire face configuration, all of its components (e.g., both eyes and the mouth in the case of vertebrates) and their spatial relationship need to be perceived (Bombari et al., 2009). The vast research on face perception has mostly covered vertebrates and includes an understanding of its ontogeny alongside its associated underlying neural mechanisms (Kobylkov and Vallortigara, 2024; Johnson et al., 1992; Parr, 2011; Rosa-Salva et al., 2010; Taubert et al., 2020). Evidence of such a skill has been found in arthropods, specifically in social insects.

### 3.1 Face recognition by honeybees

Honeybee colonies are composed of tens of thousands of related individuals, who are distinguished from members of other colonies mostly through olfactory cues (Kalmus and Ribbands, 1952; Mann and Breed, 1997). Moreover, honeybees do not exhibit distinct facial patterns and do not display face recognition within their species, meaning that honeybees may have not undergone adaptive pressures to recognize faces specifically, at least in the context of kin recognition. These characteristics should disqualify bees as candidates for the presence of a dedicated face-perception system. However, as for the other perceptual skills described in this review, perceiving living-looking objects may be a core skill of animals, without the requirement of a specific social need for it. As stated above, the configuration of face-like stimuli is virtually universal. If a dedicated face-perception system can be found in honeybees’ brains, this would suggest that its development may not require a specific evolutionary pressure but is instead a widespread skill across taxa.

The first evidence of such a skill comes from research from Dyer et al. (2005). Here, honeybees were presented with two images. The first depicted a specific human face and was associated with a food reward (sugar water). The second instead depicted a schematic face (basic geometric shapes: two circles, a triangle, and a straight horizontal line representing eyes, nose, and mouth respectively), and was associated with a punishment (bitter solution). The bees successfully learned to discriminate between the two stimuli, choosing the human face even in the absence of the reward. Moreover, when asked to select between the learned human face and a never-before-seen one, the bees maintained a preference for the former, demonstrating that they learned the specific individual. Crucially, however, if both the familiar and the novel face were rotated by 180°, the bees’ performance dropped at chance level. The study by Dyer et al. (2005) focuses on the learning and discrimination of a specific human face, which may be processed with any generalized circuit of visual perception. As such, it cannot say much about the usage of face-like configurations to detect animated objects. However, the performance drop observed in the last condition may suggest that the bees learned the stimulus in its configuration, rather than focusing on specific local cues, which is one of the key characteristics of face-detection circuitry in vertebrates (Parr, 2011). It cannot be excluded however that the bees were still only using the presence of specific local cues on specific sections of the stimuli, which would instead disqualify this as evidence for a generalized animacy cue detector.

Discerning evidence on the topic comes from Avarguès-Weber et al. (2010). Here, the authors presented honeybees with schematic, face-like patterns (two circles for the eyes, one short rectangle for the nose, and a long rectangle for the mouth). Notably, the bees were not exposed to a single stimulus but could visit different schematic faces where the distance between the elements varied. Concurrently, the bees were also exposed to different non-face-like patterns: these contained the same geometric figures but scrambled around, so as to not present a face configuration anymore. Half of the animals were trained to associate face-like stimuli with a reward, while the other half were trained to do so with non-face-like stimuli. After training, each bee was asked to choose between two completely novel stimuli and categorize them as either faces or non-faces. The bees were successful in learning the stimuli category, consistently choosing the correct novel stimulus at test. The authors then presented the bees with the choice between a face-like stimulus and a 180° rotated version of it. The bees trained on the face-like category consistently chose the upright stimulus, while the bees trained on the non-face-like category chose the rotated one. Avarguès-Weber et al. (2010) study excludes the usage of local cues in the discrimination task, as all the elements are identical across stimuli while only varying in their relative distances and positions. Bees must be learning the stimuli configuration to solve the task, which may qualify as a generalized face-detection circuit capable of detecting agents likely to be animated.

What remains to be tested, is whether face-like patterns are innately interesting to bees, or whether they are treated as any other visual stimulus. While it is true, in fact, that the bees are capable of recognizing faces as a unified category, they did so in the reported studies only after training. As stated in the introduction, animated objects should be innately interesting, and as such animacy cues should trigger an innate attentive response. As it stands, bees may be using a general visual discrimination strategy to solve the task (albeit based on the stimuli configuration), rather than a function of dedicated neural pathways for face detection. Future studies may inquire about the innate preference of bees for this type of pattern, and otherwise test whether these stimuli are in any way electively computed in the bees’ visual system.

### 3.2 A dedicated face recognition circuit in a tiny brain: the case of *Polistes fuscatus*

While both social Hymenoptera, the life history of *Polistes* paper wasps and the one of honeybees are profoundly different, especially in the social domain. *Polistes* colonies are composed of tens of individuals, rather than tens of thousands like honeybees. This element alone would make much more manageable for a wasp to remember individual sisters. Also, paper wasps recognize the members of the same colony through olfactory cues, but since these are shared across all the nest inhabitants, they cannot be used for individual recognition (Cini et al., 2019). However, in a wasp colony, hierarchies are established through aggressive interactions, which makes it crucial to remember individual competitors and avoid unnecessary multiple fights (Sheehan and Tibbetts, 2009). These wasps can in fact discriminate individual conspecifics, thanks to unique facial markings that make them look different (Tibbetts, 2002; Sheehan and Tibbetts, 2008). The ability to learn individual faces is however not just another visual pattern learning: when trained, *Polistes fuscatus* wasps learn images of conspecific faces faster than images of caterpillars, geometric patterns or other wasps faces (of *Polistes metricus*), showing that conspecific faces were not treated as any other visual cue (Sheehan and Tibbetts, 2011). Moreover, the recognition seems to be based on the full configuration: when antennae were removed or facial features were scrambled in the study, face recognition was significantly impaired.

Whether the face recognition ability is spread across the *Polistes* genus or if it is specific to *Polistes fuscatus* is unclear. For example, the related wasp species *Polistes dominula* also uses facial features in contexts of conflict (Cini et al., 2019). Tibbetts et al. (2021) found that these two wasps employ different strategies in face recognition: *P. dominula* uses facial features if shown faces of either wasp species, whereas *P. fuscatus* uses facial features only when discriminating *P. dominula*, but full configuration when seeing conspecifics. However, even if they employ a different strategy, *P. dominula* are still capable of discriminating individual conspecifics (Cini et al., 2019; Tibbetts et al., 2021).

The primacy of faces over other stimuli in *P. fuscatus* not only supports individual recognition in social interactions, but may also constitute the basis of animacy perception, indicating the presence of a living being rather than just a visual stimulus. However, in these studies, configural processing is observed only for conspecific faces, while heterospecific faces are treated like any other visual pattern. This suggests that for *P. fuscatus*, only conspecific faces rather than any face-like pattern are perceived as special, giving the impression of a living organism.

#### 3.2.1 Face processing requires social development

Face recognition in *P. fuscatus* has been shown to be affected by social experience. Wasps socially isolated in the first week of life failed to recognize and remember other individuals and could not distinguish between wasp face images (Tibbetts et al., 2019). In a subsequent study, wasps exposed to different types of social interaction during development showed varying levels of face recognition accuracy (Pardo-Sanchez et al., 2022). Wasps reared socially performed best in face discrimination tasks. Wasps reared seeing a neighbor through a clear wall or viewing their reflection in a mirror showed intermediate accuracy. However, mere visual exposure through photographs was insufficient, producing poor face recognition results akin to complete isolation. This highlights the importance of seeing a moving, or animated, individual early in life for developing effective face recognition.

As well as the ability itself, experience affects the type of processing employed during face recognition (Pardo-Sanchez and Tibbetts, 2023). While young wasps start by using holistic processing when discriminating faces (viewing the face as a full configuration rather than a collection of separate parts), social deprivation in the second week of life cause a shift towards processing featural (i.e., focusing on the single elements of the face). This switch in strategy does not seem however to impair learning: wasps using featural or holistic processing demonstrated a similar performance when recalling faces. As stated above, individual faces may be recognized without the need to process the full configuration, which instead is more likely employed in the face-dedicated brain circuitry.

#### 3.2.2 Neural correlates of face recognition in wasps

In a recent study, Jernigan et al. (2024) used multichannel electrophysiological recordings to explore how wasp brains respond to visual stimuli. The findings revealed strong selective responses to front-facing wasp images, indicating a preference for socially relevant, forward-facing orientations over other complex patterns or geometric shapes. This suggests that paper wasps have specialized neural mechanisms for recognizing conspecifics through the use of dedicated face cells, or “wasp cells” as the authors name them. Such selectivity was mostly found in the lateral protocerebrum and mushroom bodies, suggesting that these regions are particularly involved in processing wasp-specific visual information. The lateral protocerebrum specifically, especially near the optic glomeruli, contained a high density of units selectively responsive to both front-facing wasp shapes and colors. Additionally, some “wasp cells” selectively responded to individual facial features, such as markings or stripes.

The presence of the “wasp cell” clearly indicates a specialized circuit rather than a component of a general pattern discrimination system. The faster learning of faces compared to other visual stimuli, its dependence on social exposure during development, and the existence of a dedicated circuit confirm that this skill is specifically for recognizing individuals, rather than a general visual processing ability. Therefore, *Polistes* wasps could offer a unique model for studying the evolution of specialized face recognition systems. The practicality of controlling for face exposure from birth in wasps, makes them particularly valuable for exploring the development and function of holistic processing across different species.

### 3.3 Cues of face recognition in other arthropod species

While the only direct evidence available for a dedicated face recognition system are the aforementioned ones on social Hymenoptera, we do possess some cues that this skill may be more widespread than currently believed.

Crayfishes (Crustacea: Decapoda) are freshwater crustaceans, common across the globe. These animals are not believed to possess complex social structures, but still engage in fights and remember their opponents (Crook et al., 2004), and use this information to form hierarchies (Bovbjerg, 1953). Given these premises, der Velden et al. (2008) tested the crayfish *Cherax destructor* ability to recognize opponents based on their facial features. To start, the authors painted yellow patches on different specimens, either on their faces or on their claws. Thereafter, they selected an unpainted individual (i.e., the experimental subject), and placed the two in the same pen to interact. Lastly, the unpainted individual was placed in a new pen together with the familiar painted crayfish and a second, unfamiliar and unpainted individual. The subjects showed a preference for the familiar individual when this had a marking on their face, but showed no preference for the ones that had a marking on their claws. The authors then tested how the presence of specific natural face characteristics (i.e., the face width and color), by testing the preference of the experimental subject for the familiar individual vs. a starkly different unfamiliar individual (i.e., wide face vs. narrow face, light face vs. dark face). The authors observed that under this simplified comparison, and specifically for the width condition, the crayfishes were capable of individual recognition, without the need for facial markings. While the use of markings is undoubtedly an example of feature learning, the face remains an elective location for visual search and analysis. This explains why markings here, but not somewhere else, generates recognition, and why variations in the face are alone sufficient in triggering recognition. Such attentional primacy may constitute the core of a dedicated face circuitry.

A similar observation has been made on Jumping spiders. While these animals are not social, they do have a rich behavioral repertoire when it comes to conspecific interaction, especially exemplified in male-to-male competition and male-to-female mating dances (Jackson, 2014). Dahl and Cheng (2024) hypothesized that jumping spiders may be capable of remembering individuals they interacted with, and that they do so based only on visual cues. The authors employed a habituation-dishabituation paradigm: each spider was first placed in visual contact with a specific individual (both spiders were put in the same box, but separated from each other through a glass partition), and left to interact. Then, the two animals were visually separated. After this separation, the spider could either be placed in visual contact with the same individual of the first interaction, or with a novel one. The spiders showed a clear rebound in interest for the novel individual, measured as the average distance maintained from the glass partition. While this study cannot speak to the cue used by the animals to perform the discrimination, the presence of such a rapid learning in a social context suggests the presence of a dedicated “spider recognizer” circuit.

The possibility that such a spider recognizer mechanism is linked to the same configural face perception system found in other animals is supported by a recent study by Rößler et al. (2022). The authors placed *Salticus scenicus* spiders on a raised platform, taping to a trapezoidal shape. After a gap, a second platform was present, with one of 5 stimuli placed in its center. All of the stimuli were designed to depict another jumping spider species. Given the small size of *S. scenicus*, these spiders are often preyed upon by bigger salticids, and as such the stimuli were supposed to constitute a potential threat to survival. Two of the stimuli were dead specimens of a mimetic or non-mimetic jumping spider species (*Marpissa muscosa* and *Phidippus audax* respectively). The other three were high-resolution 3D prints, one reproducing a *P. audax*, one being an ellipsoid blob of the same volume, and the last being an identical ellipsoid, but with four of the spider eyes reproduced on its “face.” The authors recorded the minimum distance reached from the stimulus and the probability to freeze and escape. Unsurprisingly, the authors observed that spiders quickly froze and fled when faced with the 3D printed spider model and the dead *P. audax*, showed mixed response to the mimetic *M. muscosa*, while had no observable reaction to the blob. Crucially, while the blob with eyes did not cause a reaction identical to the spider model, the spider still showed an increased amount of freezing and maintained a further distance from it in respect to the eyeless blob. To further inquire on the effect of the eyes in the recognition process of jumping spiders, the author designed a sixth stimulus: identical to the 3D printed *P. audax* model, but with the eyes removed. In this case, the spider maintained a distance from the stimulus comparable with the full spider model, but spent a significantly higher time in freezing. This evidence altogether suggests that while the eyes are not the central cue to determine the spider behavior, they are fundamental element in the decision-making process, probably causing an initial trigger for more detailed scanning to follow. In this perspective, the eye positioning may constitute the basis of the spider’s face. Although more eyes are present than in vertebrate faces, the general triangular and top-heavy configuration is maintained, which may suggest a wide universality of the face detection pattern.

The evidence discussed in the last paragraph attests to the predisposition of arthropods to attend to faces and face-like stimuli, and at least in the case of paper wasps even describe a neuronal population dedicated to the task. As for the previously discussed motion animacy cues, the behaviors and preferences are very similar to what is observed in the vertebrate literature (Kobylkov and Vallortigara, 2024; Rosa-Salva et al., 2010; Valenza et al., 1996), crucially including the inversion effect (Yin, 1969). The presence of this effect is in our opinion particularly surprising, as arthropods can crawl upside-down and as such are not bound to the upright position as most vertebrates are. It is possible that the advantages of being able to recognize faces as rotation invariant may not match the computational cost required, which would make the skill unfavored by natural selection. On the other hand, it is also possible that crawling arthropods meet each other most frequently face-to-face while on the same plane, and as such equally oriented. Regardless of the reason, the similarity of the mechanism between arthropods and vertebrates suggests the presence of a similar neural substrate. Future studies may look for “face areas” outside of social Hymenoptera, cementing the idea that such a skill is specific and widespread across taxa.

## 4 The elephant in the room: thanatosis

A huge variety of arthropods engage in “feigning death” behavior (Humphreys and Ruxton, 2018). Thanatosis, often termed tonic immobility, describes the sudden interruption of movement of an animal (Rogers and Simpson, 2014). This does not limit to freezing, being accompanied by a complete loss of control over the body, with the animal curling up, protruding the tongue, etc. The animal does not just stop moving, it actually looks dead. If animacy is the property of appearing alive, and it is the characteristics that predators may exploit while hunting, looking dead is the perfect countermeasure (Gonçalves and Biro, 2018). Extravagant cases may employ movement to actively hide animacy cues and escape danger. For example, the mimetic insect *Extatosoma tiaratum* will actively swing its body in the presence of wind, mimicking the oscillatory movement of leaves and branches to hide its motion animacy signals (Bian et al., 2016).

Thanatosis has been described in a massive variety of invertebrates (Humphreys and Ruxton, 2018), and it is mostly used by animals as a last resort when hunted. Frequently, the fooled predator is a vertebrate (Gyssels and Stoks, 2005; O’Brien and Dunlap, 1975; Moore and Williams, 1990; King and Leaich, 2006). However, many thanatosis displays are performed to defend from other arthropods. For example, gynes of the stingless bee *Melipona beecheii* have been observed to escape aggression from workers by death feigning (van Veen et al., 1999). In these events, workers ceased their attack and carried the gyne to the colony waste dump, as they would do with any cadaver. While we do not know which cue the worker bees were using to tell that the attacked gyne died, it appears that the sudden tonic immobility stripped away the animacy appearance, suggesting a reliance on such a cue. A similar behavior has been observed in fire ants (Cassill et al., 2008), where especially young individuals are likely to feign death when attacked by conspecific of rival colonies. The ant reliance on death (and live) cues is also exploited by co-evolved antagonistic species: the guest beetle *Claviger testaceus* feigns death in order to get transported inside *Lasius flavius* anthills, mistaken for a fresh food source. Once inside, the beetle can freely prey upon the ants’ eggs, larvae and pupae (Cammaerts, 1999). Males *Pisaura mirabilis* spiders feign death during mating, along with providing the female with a nuptial gift (Hansen et al., 2008). This behavior increased significantly copulation success, decreasing cannibalistic attacks of the female (Bilde et al., 2005). The examples of the efficacy of thanatosis when directed towards arthropods are almost endless, for which reason we cannot provide a full account of it as part of this review. What is certain, is that for every example of thanatosis behavior, there must be a specific cue on which the predator relied on that the prey successfully suppressed. We hope that more studies will try to approach the topic of thanatosis from the recipient perspective, as we believe that many of those behaviors may be rooted in animacy perception.

## 5 Conclusion

In this review, we have discussed a substantial amount of literature relevant to animacy perception. We acknowledge that there may be many other examples that we failed to describe, focusing on other species and other behavioral contexts that are well known in the natural sciences literature but that have never been reported as part of the topic of the animacy perception, which is mainly the domain of cognitive sciences. Nevertheless, we hope that this review will ignite interest and inspire readers and researchers to reevaluate and contextualize the existing evidence, thereby broadening their perspective and encouraging further exploration in this field.
